# Effect of Intraoperative Magnesium Sulfate Administration on Blood Glucose Control following Total Joint Arthroplasty in Patients with Diabetes

**DOI:** 10.3390/jcm11113040

**Published:** 2022-05-27

**Authors:** Jin-Woo Park, Eun-Kyoung Kim, Jiyoun Lee, Seung Hyun Chung, Gihong Boo, Sang-Hwan Do

**Affiliations:** 1 Department of Anesthesiology and Pain Medicine, Seoul National University Bundang Hospital, Seongnam 13620, Korea; jinul8282@gmail.com (J.-W.P.); heykiki1@hanmail.net (J.L.); book2909@naver.com (G.B.); 2 Department of Anesthesiology and Pain Medicine, College of Medicine, Seoul National University, Seoul 03080, Korea; eunkyoung2lovely@gmail.com; 3 Department of Anesthesiology and Pain Medicine, Uijeongbu Eulji Medical Center, Eulji University, Uijeongbu 11759, Korea; anejsh@naver.com

**Keywords:** arthroplasty, blood glucose, diabetes mellitus, insulin, magnesium sulfate, postoperative pain

## Abstract

Magnesium deficiency, which is known to be highly prevalent among patients with diabetes, has been associated with insulin resistance and poor glucose control. Here, we aimed to investigate the effects of intraoperative magnesium administration on postoperative glucose control in patients with diabetes. We retrospectively reviewed the medical records of patients with type 2 diabetes who had undergone total joint arthroplasty at a tertiary hospital, where intraoperative magnesium sulfate injections were frequently performed for postoperative analgesia. The patients were grouped based on whether treated with magnesium or not (magnesium vs. control groups). We investigated postoperative blood glucose levels and sliding scale insulin requirements. After propensity matching, 170 patients were allotted to each group. Both the mean glucose level and the incidence of a mean glucose level of >200mg/dL were significantly lower in the magnesium group than in the control group (*p* = 0.040 and 0.013, respectively). There was also a lower insulin requirement in the magnesium group (*p* = 0.043). Multivariate logistic regression revealed that magnesium treatment was significantly related to a less frequent incidence of a mean blood glucose level of >200 mg/dL (*p* = 0.047). This study demonstrated that magnesium sulfate infusion was associated with an improved postoperative blood glucose profile in patients with diabetes.

## 1. Introduction

Patients with diabetes have demonstrated a significantly increased risk of surgical and systemic complications following total joint arthroplasty (TJA) [1,2]. Elevated preoperative glycosylated hemoglobin A1c (HbA1c) levels and postoperative blood glucose levels have been correlated with increased risks of periprosthetic joint infection [3].

Magnesium sulfate, an N-methyl-D-aspartate antagonist, is an effective adjuvant treatment for postoperative pain. Previous studies have demonstrated that an intraoperative magnesium sulfate infusion during TJA decreases postoperative pain and opioid analgesic requirements [4,5]. In addition, magnesium is a cofactor of enzymatic pathways involved in the modulation of glucose transport across cell membranes. Hypomagnesemia is highly prevalent in patients with diabetes, and magnesium supplementation has been shown to help stabilize blood glucose levels in these patient populations [6,7,8]. Therefore, perioperative magnesium treatment may also help stabilize postoperative blood glucose levels in patients with diabetes. However, to our knowledge, there are currently no clinical studies investigating the effects of intraoperative magnesium administration on postoperative glucose control in surgical patients with diabetes.

In this retrospective analysis, we aimed to investigate the effects of intraoperative magnesium sulfate administration on postoperative blood glucose control following TJA in patients with type 2 diabetes. We hypothesized that patients administered with magnesium sulfate during surgery would show more stable blood glucose profiles after surgery.

## 2. Materials and Methods

### 2.1. Study Population

We retrospectively reviewed the electronic medical records of patients with type 2 diabetes who underwent total hip/knee arthroplasty under spinal anesthesia at the Seoul National University Bundang Hospital (SNUBH) between 2016 and 2020. The patients who did not take diabetes medication, received general or epidural anesthesia, were postoperatively admitted to the intensive care unit, had incomplete medical records, or had contraindications to intraoperative magnesium sulfate treatment (severe renal insufficiency, bradycardia, atrioventricular block ≥ 2nd degree, and heart failure) were excluded from the study [9,10]. If a patient underwent multiple TJAs during the study period, only the first case was included in this analysis. 

### 2.2. Anesthetic Management

In the preoperative holding area, the patients were premedicated with intravenous midazolam. To perform spinal anesthesia, an optimal dose of 0.5% hyperbaric bupivacaine or 0.75% levobupivacaine with 10–20 µg fentanyl was intrathecally injected. For the patients who requested sedation, after confirming that the spinal sensory block height was adequate and the vital signs were stable, intravenous propofol or dexmedetomidine was administered at the discretion of the attending anesthesiologist. A responsiveness score of 3 or 4, using the modified observer’s assessment of Alertness/Sedation scale, was maintained during the operation [11]. Additionally, it was at the anesthesiologist’s preference whether to intravenously administer magnesium sulfate or not. In SNUBH, intraoperative magnesium sulfate infusions were frequently administered as an adjuvant for postoperative analgesia. Some attending anesthesiologists in our orthopedic surgery unit routinely administered magnesium sulfate during TJA, but the others did not; except for contraindications to magnesium administration, there was no specific patient condition that affected the practice. The administration regimen was as follows: during anesthesia induction, magnesium sulfate was loaded over 15 min (50 mg·kg^−1^) and then continuously infused at a rate of 15 mg·kg^−1^·h^−1^ until the end of the surgery. Postoperatively, intravenous, patient-controlled analgesia (PCA) was initiated and continued until postoperative day 3. Based on the patient’s age, weight, and comorbidities, intravenous PCA comprised 8–15 μg·mL^−1^ fentanyl within a total volume of 100 mL and was administered as 1 mL bolus doses with 10 min lockout intervals and without basal continuous infusion.

### 2.3. Study Outcomes and Data Collection

In SNUBH, the postoperative blood glucose levels of patients with diabetes were routinely monitored every 4 h following TJA and were transferred to the electronic medical records. Point-of-care testing devices with a glucose dehydrogenase-based system were utilized, and blood samples were obtained by pricking the patient’s finger. Diabetic medication for each patient was resumed on POD 1 and a normal diet was also. Sliding scale lispro insulin was administered when levels exceeded 200 mg/dL during the postoperative period. The primary outcome of this retrospective study was the mean postoperative blood glucose level during postoperative days 0 to 2. We also determined the quantities of insulin required to manage postoperative hyperglycemia until postoperative day 2. The quantity of opioids used for PCA and rescue treatment for postoperative pain management was summed and calculated as a morphine equivalent dose. Demographic data; the American Society of Anesthesiologists’ physical status classification scores; data on preoperative comorbidities, hematocrit levels, HbA1c levels, blood glucose levels, and insulin medication; and operational data on operation time, estimated blood loss, intraoperative blood pressure and fluid, sedation medication, years at surgery, and midazolam premedication were collected.

### 2.4. Statistical Analysis

The patients were grouped according to whether or not magnesium sulfate was administered during surgery: the magnesium group and the control group. Continuous variables were expressed as medians with interquartile ranges and categorical variables as numbers (%). The Mann–Whitney U-test was used to compare continuous variables between the two groups, and the chi-square or Fisher’s exact test was used to compare categorical variables, as appropriate. 

To minimize the risk of confounding bias between the magnesium and control groups, propensity score matching was performed at a 1:1 ratio using the nearest-neighbor method. The matching included all the covariates of the demographic, comorbidities, preoperative laboratory values, and operational data. The propensity scores were calculated using a logistic regression analysis. To confirm the balance between the groups, the standardized mean difference was tested at <0.1 for each covariate.

To determine the association between a mean postoperative glucose level of >200 mg/dL and each variable, a univariate logistic regression was performed. Variables with a *p*-value of <0.2 from the univariate regression analysis were included in the multivariate logistic regression model. The Statistical Package for Social Sciences software version 21.0 (SPSS Inc., IBM, Chicago, IL, USA) was used for all statistical analyses. A two-sided *p*-value of <0.05 was considered statistically significant.

## 3. Results

**Figure 1:**
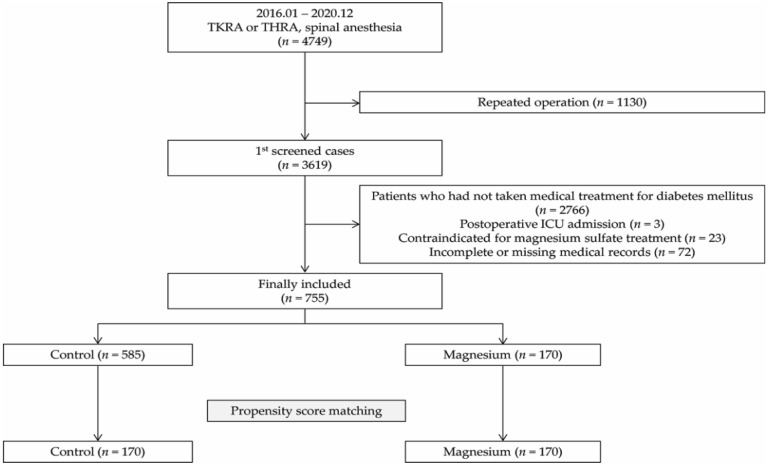
Flow chart of patient selection. ICU, intensive care unit.

A total of 4749 patients underwent TJA at SNUBH between 2016 and 2020, of whom 755 patients were included in the final analysis (control group, 585; magnesium group, 170). After the propensity score matching, 170 patients were allotted to each group (Figure 1). 

The baseline characteristics before and after the propensity score matching are shown in Table 1. The standardized mean differences of all the covariates were <0.1, which confirmed that the match balance was adequate. Of the 755 patients, the mean postoperative blood glucose level was significantly lower in the magnesium group than in the control group (*p* = 0.022; Table 2). The incidence of a mean postoperative glucose level of >200 mg/dL was lower in the magnesium group than in the control group (odds ratio, 0.62; 95% confidence interval (CI), 0.42–0.91; *p* = 0.015). The Lispro insulin required to manage postoperative hyperglycemia was also less in the magnesium group (odds ratio, 0.67; 95% CI, 0.47–0.95; *p* = 0.024). Patients in the magnesium group had a lower opioid requirement than those in the control group (*p* = 0.004). We obtained similar results after the propensity score matching. The mean postoperative blood glucose level was lower in the magnesium group than in the control group (*p* = 0.040; Table 2). The incidence of a mean postoperative glucose level of >200 mg/dL was also lower in the magnesium group (odds ratio, 0.55; 95% CI, 0.34–0.88; *p* = 0.013). Further, the magnesium group had a lower requirement for postoperative lispro insulin (odds ratio, 0.63; 95% CI, 0.41–0.99; *p* = 0.043) and a lower opioid requirement than that of the control group (*p* = 0.003).

Multivariate logistic regression analysis showed that intraoperative magnesium treatment (odds ratio, 0.64; 95% CI, 0.41–0.99; *p* = 0.047) and preoperative hematocrit (odds ratio, 0.91; 95% CI, 0.88–0.96; *p* < 0.001), HbA1c (odds ratio, 2.45; 95% CI, 1.95–3.07; *p* < 0.001), and blood glucose levels (odds ratio, 1.02; 95% CI, 1.02–1.03; *p* < 0.001) were significantly associated with the incidence of a mean postoperative blood glucose level of >200 mg/dL (Table 3). The multivariate model showed a good fit (Hosmer–Lemeshow test *p* = 0.396).

In the propensity-matched cohort, there were 29 patients with unsatisfactory preoperative glycemic control (HbA1c ≥ 8.0). Sixteen patients were in the control group, and thirteen were in the magnesium group; the baseline data were not significantly different between the two groups (Table S1). In the subgroup analysis of these patients, there was a marked difference in the mean postoperative glucose between the control and magnesium groups [185.4 (175.5–221.8) vs. 243.8 (208.5–265.8); *p* = 0.022; Table 4]. The magnesium group also showed a lower incidence of a mean glucose level of >200 mg/dL (odds ratio, 0.21; 95% CI, 0.04–1.02; *p* = 0.047) than that of the control group.

## 4. Discussion

This retrospective study demonstrated that intraoperative magnesium sulfate administration was significantly associated with lower glucose levels and reduced insulin and analgesic requirements after surgery. Propensity score matching strengthened the analysis, and multivariate logistic regression showed that intraoperative magnesium was a significant factor influencing glucose stability. To our knowledge, this is the first study to report on the effect of intraoperative magnesium treatment on postoperative glucose control. 

Surgical stress can adversely affect glycemic control and may result in postoperative hyperglycemia among patients with and without diabetes. During the perioperative period, stress responses may induce sympathetic stimulation and subsequently raise stress hormones such as catecholamine, cortisol, glucagon, and growth hormone levels, thereby increasing endogenous glucose production [12,13,14]. Insulin secretion is also inhibited, thus promoting insulin resistance [15]. In addition, preoperative starvation is also known to increase postoperative stress and lead to the risk of hyperglycemia [16,17]. Postoperative hyperglycemia has been associated with a variety of unfavorable outcomes such as increased mortality, surgical site infection, acute kidney injury, and cardiovascular complications [18,19]. Therefore, the prediction and prevention of hyperglycemia are of great clinical importance.

Magnesium is one of the essential inorganic nutrients; thus, we have to obtain magnesium directly from diet or through supplementation. During the perioperative period, hypomagnesemia is common but seldom recognized [20]. A previous study reported that the incidence of hypomagnesemia was 19% preoperatively, 71% immediately after cardiac surgery, and decreased to 66% at 24 h postoperatively [21]. Furthermore, magnesium levels are frequently lower in patients with diabetes than in those without. Additionally, magnesium supplementation is known to improve insulin sensitivity and metabolic control in patients with diabetes [6,22,23,24]. Therefore, according to the results of this study, intraoperative magnesium sulfate administration should have helped to increase blood magnesium concentrations, avoid hypomagnesemia, and potentially assist with glucose control in patients with diabetes. According to our previous studies, the magnesium sulfate regimen used in this study increased the magnesium level up to approximately 1.15–1.60 mM immediately after surgery without the complication of hypermagnesemia, and it decreased to within a normal range within 24 h after surgery [4,25,26,27,28].

Multivariate regression analysis revealed that intraoperative magnesium treatment and a preoperative HbA1c/glucose level were determinants of a high postoperative mean glucose level (>200 mg/dL). Multiple previous studies have reported that a postoperative mean blood glucose level of >200 mg/dL is associated with surgical site infection in patients undergoing TJA [3,29]. Therefore, it is highly probable that intraoperative magnesium therapy would affect the incidence. A high preoperative HbA1c is also known to be one of the risk factors for periprosthetic joint infection after TJA [3,30]. In the subgroup analysis of the patients with poor glycemic control, the magnesium group showed a better postoperative blood glucose profile than the control group did, which highlighted the value of magnesium sulfate administration. Here, the effect of intraoperative magnesium sulfate on the mean postoperative blood glucose level seemed to be more pronounced in patients with higher preoperative HbA1c levels, which may be owing to hypomagnesemia being associated with poor glycemic control and more prevalent among patients with HbA1c levels of ≥8.0% [23,31].

In addition, the preoperative hematocrit was also a predisposing factor for the incidence of a postoperative mean blood glucose level of >200 mg/dL. Since TJAs inevitably lead to significant postoperative bleeding, hypovolemia or hemodilution could occur in the early postoperative period and subsequently increase sympathetic autonomic responses. Low basal hematocrit levels could aggravate acute hemodilutional anemia and further sympathetic activation, resulting in a higher risk of postoperative hyperglycemia [18,32]. 

Magnesium is well-known to have an antinociceptive effect and attenuate a stress response [33]. Numerous studies have reported that an intraoperative magnesium infusion reduced the intraoperative anesthetic requirements and postoperative analgesic use [4,25]. In a previous randomized controlled trial, intraoperative magnesium infusion was significantly associated with a decreased cortisol level [34]. By reducing the stress response, magnesium treatment could provide additional assistance in obtaining glycemic control [35]. Anesthetic methods and drugs could also affect postoperative glucose profiles. Regional anesthesia has been associated with lower insulin resistance compared to general anesthesia [13,36]. For this analysis, we limited the anesthetic method to spinal anesthesia. Both intraoperative dexmedetomidine and propofol have been reported to have favorable effects on perioperative glucose stability by attenuating the stress response [37,38]. However, in our results, the incidence of a mean postoperative glucose concentration of >200 mg/dL was not significantly different between the patients with and without intraoperative sedation, which may be owing to the routine midazolam premedication and relatively low stress response under spinal anesthesia [13,36]. 

There are some limitations in this study. First, due to the retrospective nature of this study, the magnesium treatment during surgery was not randomized. In order to reduce selection bias, we performed propensity score matching to balance basal characteristics and operation data between the magnesium and control groups. Second, perioperative glucose management was performed at the discretion of the orthopedist in charge and was not strictly regulated. Furthermore, a systematic approach should have been more helpful in postoperative glycemic control. Strictly controlled perioperative blood glucose management using long-acting insulin and short-acting insulin confirmed a superior postoperative glucose profile than sliding scale insulin [39]. Third, we could not assess patients’ magnesium levels, which are not routinely measured perioperatively in our institution. In many previous studies of our team, the intraoperative magnesium regimen in our protocol has been shown to increase serum magnesium to a safe level [4,25,26,27,28,40]. However, magnesium infusion must be carefully decided because hypermagnesemia could cause deleterious complications such as hypotension, arrhythmia, muscle weakness, or even respiratory depression. In SNUBH, the magnesium sulfate regimen is contraindicated in patients with severe renal insufficiency, bradycardia, atrioventricular block ≥ 2nd degree, or heart failure. Meanwhile, it is still unknown whether the intraoperative magnesium treatment would help to stabilize postoperative glucose control just by relieving the deficiency or cause favorable effects in patients with normal magnesium levels as well. Further prospective studies should be required to address this issue. Finally, the generalizability of this study may be limited because we retrospectively analyzed data from a single medical center.

## 5. Conclusions

This retrospective analysis demonstrated that intraoperative magnesium sulfate administration was associated with an improved postoperative glucose profile and reduced analgesic requirements following TJA. This finding is of great clinical value because it provides additional information for intraoperative magnesium treatment to improve postoperative recovery. However, our results were obtained from retrospective chart review and should be confirmed in further prospective trials.

## Figures and Tables

**Table 1:** Baseline characteristics before and after propensity score matching.

	Unmatched Cohort (*n* = 755)		SMD	*p*	Matched Cohort (*n* = 340)		SMD	*p*
	Control *n* = 585	Mg *n* = 170			Control *n* = 170	Mg *n* = 170		
Age, year	71.0 (65.0–75.0)	70.5 (65.0–74.8)	0.079	0.558	70.0 (65.0–76.0)	70.5 (65.0–74.8)	0.055	0.721
Sex								
Male	124 (21.2%)	41 (24.1%)	0.070	0.417	45 (26.5%)	41 (24.1%)	0.054	0.618
Female	461 (78.8%)	129 (75.9%)			125 (73.5%)	129 (75.9%)		
BMI, kg m^−2^	26.9 (24.5–29.8)	26.6 (24.3–28.9)	0.111	0.185	26.1 (24.0–29.2)	26.6 (24.3–28.9)	0.018	0.836
ASA status (II/III)								
II	456 (77.9%)	134 (78.8%)	0.021	0.808	128 (75.3%)	134 (78.8%)	0.084	0.439
III	129 (22.1%)	36 (21.2%)			42 (24.7%)	36 (21.2%)		
Hypertension	451 (77.1%)	126 (74.1%)	0.069	0.421	128 (75.3%)	126 (74.1%)	0.027	0.803
Ischemic heart disease	77 (13.2%)	18 (10.6%)	0.080	0.373	19 (11.2%)	18 (10.6%)	0.019	0.862
Cerebrovascular disease	41 (7.0%)	8 (4.7%)	0.098	0.283	10 (5.9%)	8 (4.7%)	0.053	0.628
Preoperative Hematocrit, %	39.6 (37.1–42.4)	40.1 (37.6–42.5)	0.144	0.171	39.9 (37.5–42.7))	40.1 (37.6–42.5)	0.086	0.723
Preoperative HbA1c, %	6.7 (6.2–7.3)	6.6 (6.1–7.2)	0.111	0.119	6.6 (6.2–7.3)	6.6 (6.1–7.2)	0.010	0.535
Preoperative blood glucose level, mg/dL	129.0 (111.0–150.0)	129.0 (112.3–146.0)	0.010	0.963	132.0 (111.0–151.0)	129.0 (112.3–146.0)	0.056	0.800
Insulin medication	27 (4.6%)	5 (2.9%)	0.088	0.340	6 (3.5%)	5 (2.9%)	0.033	0.759
Type of surgery								
TKRA	465 (79.5%)	119 (70.0%)	0.220	0.009	126 (74.1%)	119 (70.0%)	0.092	0.398
THRA	120 (20.5%)	51 (30.0%)			44 (25.9%)	51 (30.0%)		
Operative characteristics								
Operation time, min	130.0 (105.0–150.0)	135.0 (115.0–153.8)	0.221	0.020	135.0 (115.0–155.0)	135.0 (115.0–153.8)	0.010	0.716
Estimated blood loss, mL	100.0 (70.0–250.0)	150.0 (70.0–550.0)	0.284	< 0.001	115.0 (70.0–350.0)	150.0 (70.0–550.0)	0.049	0.177
Intravenous fluid, mL	450.0 (300.0–700.0)	500.0 (300.0–950.0)	0.208	0.087	500.0 (350.0–800.0)	500.0 (300.0–950.0)	0.064	0.776
MBP, mmHg	74.5 (70.1–80.8)	73.2 (68.1–80.7)	0.110	0.141	74.5 (69.5–81.6)	73.2 (68.1–80.7)	0.097	0.338
Sedation								
None	249 (42.6%)	84 (49.4%)	0.999	< 0.001	85 (50.0%)	84 (49.4%)	0.077	0.777
Dexmedetomidine	96 (16.4%)	76 (44.7%)			72 (42.4%)	76 (44.7%)		
Propofol	240 (41.0%)	10 (5.9%)			13 (7.6%)	10 (5.9%)		
Years at surgery								
2016–2018.6	316 (54.0%)	73 (42.9%)	0.223	0.011	66 (38.8%)	73 (42.9%)	0.084	0.440
2018.7–2020	269 (46.0%)	97 (57.1%)			104 (61.2%)	97 (57.1%)		
Premedication								
Midazolam, mg	2.0 (0.5–3.0)	1.5 (0.5–3.0)	0.125	0.098	1.5 (0.5–3.0)	1.5 (0.5–3.0)	0.032	0.779

Data are median [IQR] or number (%). SMD, standardized mean difference; BMI, body mass index; ASA, American Society of Anesthesiologists; HbA1c, glycosylated hemoglobin A1c; TKRA, total knee replacement arthroplasty; THRA, total hip replacement arthroplasty; MBP, mean blood pressure.

**Table 2:** Mean postoperative blood glucose level, insulin requirement, and postoperative analgesia during postoperative days 0 to 2, before and after propensity score matching.

	Control	Mg	Odds Ratio (95% CI)	*p*
Before matching				
Blood glucose level				
Mean	183.0 (157.8–210.7)	176.8 (153.1–198.8)		0.022
Mean > 200 mg/dL	195/585 (33.3%)	40/170 (23.5%)	0.62 (0.42–0.91)	0.015
Postoperative insulin requirement	392/585 (67.0%)	98/170 (57.6%)	0.67 (0.47–0.95)	0.024
MEC, mg	140.0 (99.5–188.0)	123.5 (83.5–175.5)		0.004
				
After matching				
Blood glucose level				
Mean	184.2 (157.6–213.8)	176.8 (153.1–198.8)		0.040
Mean > 200 mg/dL	61/170 (35.9%)	40/170 (23.5%)	0.55 (0.34–0.88)	0.013
Postoperative insulin requirement	116/170 (68.2%)	98/170 (57.6%)	0.63 (0.41–0.99)	0.043
MEC, mg	140.0 (94.0–198.0)	123.5 (83.5–175.5)		0.003

Data are median [IQR] or number (%). MEC, morphine equivalent consumption.

**Table 3:** Results of multivariate analysis of variables associated with mean postoperative blood glucose > 200 mg/dL.

	Odds Ratio (95% CI)	*p*
Magnesium continuous infusion	0.64 (0.41–0.99)	0.047
ASA		
II	1	
III	1.04 (0.67–1.62)	0.857
HTN	1.21 (0.78–1.87)	0.393
CVD	1.38 (0.67–2.85)	0.379
Preoperative hematocrit	0.91 (0.88–0.96)	<0.001
Preoperative HbA1c	2.45 (1.95–3.07)	<0.001
Preoperative blood glucose level	1.02 (1.02–1.03)	<0.001
Insulin medication	0.91 (0.38–2.16)	0.823
Estimated blood loss	1.00 (1.00–1.00)	0.758
Intravenous fluid	1.00 (1.00–1.00)	0.890
Sedation		
None	1	
Dexmedetomidine	1.02 (0.64–1.63)	0.928
Propofol	0.71 (0.46–1.08)	0.105

Data are median [IQR] or number (%). ASA, American Society of Anesthesiologists; HTN, Hypertension; CVD, Cerebrovascular disease; HbA1c, glycosylated hemoglobin A1c.

**Table 4:** Mean postoperative blood glucose level and insulin requirement among the propensity-matched patients with preoperative HbA1c ≥ 8.0.

	Control (*n* = 16)	Mg (*n* = 13)	Odds Ratio (95% CI)	*p*
Blood glucose level				
Mean	243.8 (208.5–265.8)	185.4 (175.5–221.8)		0.022
Mean > 200 mg/dL	12 (75%)	5 (38.5%)	0.21 (0.04–1.02)	0.047
Postoperative insulin requirement	16 (100%)	12 (92.3%)		0.448

Data are median [IQR] or number (%). HbA1c, glycosylated hemoglobin A1c.

## Data Availability

The datasets used and/or analyzed during the current study are available from the corresponding author on reasonable request.

## Supplementary Materials

The following supporting information can be downloaded at: https://www.mdpi.com/article/10.3390/jcm11113040/s1, Table S1: Baseline characteristics of the patients with unsatisfactory glycemic control (HbA1c ≥ 8.0; the propensity-matched cohort).
