# Supplementary material for: Effect of Intraoperative Magnesium Sulfate Administration on Blood Glucose Control following Total Joint Arthroplasty in Patients with Diabetes
Source: J Clin Med. 2022 May 27;11(11):3040. doi: 10.3390/jcm11113040 (PMC9181658; doi:10.3390/jcm11113040)
Supplement: Supplementary file 1 [file jcm-11-03040-s001.zip › jcm-1614112-supplementary.pdf]

Table S1. Baseline characteristics of the patients with unsatisfactory glycemic control (HbA1c ≥ 8.0; the propensity matched cohort)

	**Control (n = 16)**	**Mg (n = 13)**	***P***
Age, year	66.5 (61.0-73.3)	69.0 (58.0-72.0)	0.871
Sex			
Male	4 (25.0%)	6 (46.2%)	0.270
Female	12 (75.0%)	7 (53.8%)	
BMI, kg m^{-2}	26.2 (25.1-27.7)	30.2 (24.9-31.9)	0.110
ASA status (II/III)			
II	6 (37.5%)	5 (38.5%)	1.000
III	10 (62.5%)	8 (61.5%)	
Hypertension	12 (75.0%)	13 (100.0%)	0.107
Ischemic heart disease	1 (6.3%)	2 (15.4%)	0.573
Cerebrovascular disease	0 (0.0%)	2 (15.4%)	0.192
Preoperative Hematocrit, %	43.1 (39.5-45.2)	41.7 (38.2-43.7)	0.380
Preoperative HbA1c, %	8.2 (8.1-8.6)	8.6 (8.2-9.0)	0.143
Preoperative blood glucose level, mg/dL	141.0 (115.3-178.5)	137.0 (111.0-152.0)	0.495
Insulin medication	2 (12.5%)	1 (7.7%)	1.000
Type of surgery			
TKRA	10 (62.5%)	10 (76.9%)	0.454
THRA	6 (37.5%)	3 (23.1%)	
Operative characteristics			
Operation time, min	142.5(125.0-161.3)	125.0 (105.0-135.0)	0.322
Estimated blood loss, mL	125.0 (70.0-450.0)	100.0 (70.0-350.0)	0.585
Intravenous fluid, mL	525.0 (337.5-1085.0)	350.0 (300.0-800.0)	0.226
MBP, mmHg	8.19 (73.5-84.6)	73.8 (70.3-78.9)	0.351
Sedation			
None	9 (56.3%)	8 (61.5%)	0.907
Dexmedetomidine	5 (31.3%)	4 (30.8%)	
Propofol	2 (12.5%)	1 (7.7%)	
Years at surgery			
2016-2018.6	3 (18.8%)	5 (38.5%)	0.406
2018.7-2020	13 (81.2%)	8 (61.5%)	
Premedication			
Midazolam, mg	1.5 (1.0-2.3)	2.0 (0.5-3.0)	0.903
